# Improved catalytic efficiency, thermophilicity, anti-salt and detergent tolerance of keratinase KerSMD by partially truncation of PPC domain

**DOI:** 10.1038/srep27953

**Published:** 2016-06-14

**Authors:** Zhen Fang, Juan Zhang, Guocheng Du, Jian Chen

**Affiliations:** 1 Key Laboratory of Industrial Biotechnology, Ministry of Education, Jiangnan University, Wuxi 214122, China; 2 Synergetic Innovation Center of Food Safety and Nutrition, Wuxi 214122, China; 3 School of Biotechnology, Jiangnan University, Wuxi 214122, China; 4 Key Laboratory of Carbohydrate Chemistry and Biotechnology, Ministry of Education, Jiangnan University, Wuxi 214122, China; 5 National Engineering Laboratory for Cereal Fermentation Technology, Jiangnan University, Wuxi 214122, China

## Abstract

The keratinase from *Stenotrophomonas maltophilia* (KerSMD) is known for its high activity and pH stability in keratin degradation. However, catalytic efficiency and detergent tolerability need to be improved in order to be used for industrial application. In this work, we obtained several keratinase variants with enhanced catalytic efficiency, thermophilicity, and anti-salt and detergent tolerability by partially truncating the PPC domain of KerSMD. The variants all showed improved catalytic efficiency to synthetic substrate AAPF, with the V355 variant having the highest *k*_*cat*_ /*K*_*m*_ value of 143.6 s^−1^ mM^−1^. The truncation of keratinase had little effect on alkaline stability but obviously decreased collagenase activity, developing its potential application in leather treatment. The variants V380, V370, and V355 were thermophilic, with a 1.7-fold enhancement of keratinlytic activity at 60 °C when compared to the wild type. The entire truncation of PPC domain obtained the variant V355 with improved tolerance to alkalinity, salt, chaotropic agents, and detergents. The V355 variant showed more than a 40% improvement in activity under 15% (w/v) NaCl or 4% (w/v) SDS solution, showing excellent stability under harsh washing and unhairing conditions. Our work investigated how protein engineering affects the function of PPC domain of KerSMD.

Since biocatalysts are environmentally friendly and necessary for sustainable development, commercial enzymes are extensively used in the pharmaceutical, leather, and textile industry, as well as in the biotreatment for food^1^,^2^,^3^. Enzymes abundant in nature have been isolated and used for these industrial applications, however, their poor tolerance toward pH, temperature, organic solvents, salt, and abstergent and oxidative reagents drastically restrict their catalytic performance and application^4^,^5^,^6^,^7^. Therefore, increasing attention is being paid to find high performance enzymes that can adapt to extreme environments as well as possessing a wide substrate specificity.

Protein engineering strategies, such as site-directed mutagenesis and directed evolution, are always used to create excellent enzymes^8^,^9^,^10^. Those strategies are popular in the field of biotechnology, however, disadvantages still exist. Site-directed mutagenesis should be based on the crystal structure of the protein being engineered, while directed evolution needs a random mutant library for high-throughput screening^10^,^11^,^12^. Those two strategies can be time-consuming and ultimately fruitless. Therefore, it is necessary to establish a simple bio-technique for molecular modification.

It was found that some enzymes, such as amylases and dextranase, have unnecessary amino acids or domains at the C-terminus, and its truncation mutants have shown improved catalytic efficiency as well as other enzymatic properties^13^,^14^. Recently, we found that a few proteases have Bacterial Pre-peptidase C-terminal domains (PPC domains)^15^,^16^,^17^. The PPC domain always has a single alpha-helix packed against an antiparallel beta-sheet to form beta-strands. For example, the deleted C-terminal mutants of the halophilic protease MCP-03 showed that C-terminal domain was unnecessary for enzyme secretion but could improve catalytic efficiency^18^. Another example of this is the C-terminal truncation of the alkaline protease HP70 enhancing the specific activity to suc-AAPF-pNA nearly 4-fold^19^. Therefore, C-terminal truncation to improve enzyme catalytic efficiency seems like a viable strategy.

Keratinase is an excellent enzyme for feather degradation, textile treatment, leather depilation, and as well as several other industrial application^20^. In our previous work, we isolated a novel keratinase KerSMD and conducted heterologous expression in *Escherichia coli* successfully^21^,^22^. KerSMD shows great potential in hydrolyzing keratin material, but low catalytic activity, broad substrate selectivity, and bad tolerability to salt and detergent may prevent its industrial application. Fortunately, protein engineering may be a powerful method to turn the situation around^4^. Our recent work showed that KerSMD has an independent C-terminal domain and its truncation or replacement could affect enzymatic properties^22^. Therefore, studies related to the function of the C-terminal domain should be investigated.

In this work, we constructed a library of keratinase KerSMD mutants by partial truncation of C-terminal domain. All variants were successfully expressed in *E. coli*, and their biochemical properties (*e.g*., expression quantity, substrate specificity, kinetic parameters, thermostability, alkaline stability, salt stability, and detergent tolerance) were characterized. These findings not only shine light on the function of the C-terminal domain of KerSMD but also provided a useful strategy for protein engineering to improve enzyme specific activity and catalytic efficiency.

## Results and Discussion

### Construction and expression of KerSMD and its truncated mutants

**Figure 1:**
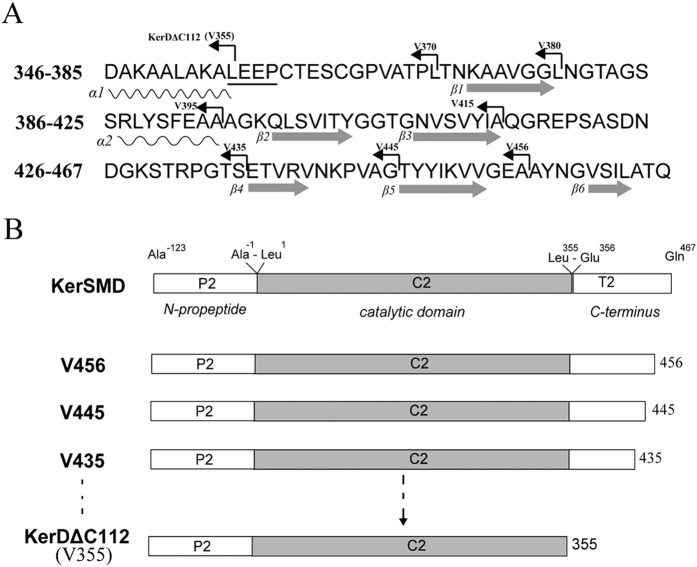
Amino acid sequence of the C-terminal domain (**A**) and schematic representation of the primary structures of KerSMD and its variants (**B**). The secondary structure of C-terminal domain was predicted by Discovery Studio 2.5. The wavy line means the α-helix and gray arrow is the β-fold. Every C-terminal deletion variant was showed by a turning arrow.

There is a special C-terminal domain (PPC domain) with a size of about 100 amino acids found in keratinase KerSMD. From our previous report, we have discussed its potential function to enhance substrate specificity of insoluble substrate^22^. In this work, the secondary structure of PPC domain was predicted (Fig. 1A). We could see that the secondary structure of the C-terminal domain consisted mainly of six β-folds (β-fold1, β-fold2, β-fold3, β-fold4, β-fold5, and β-fold6) which might be important to protein stability. To further study the roles of that domain, sites among the β-fold were chosen for truncation and mutants of KerSMD were constructed as shown in Fig. 1B.

**Figure 2:**
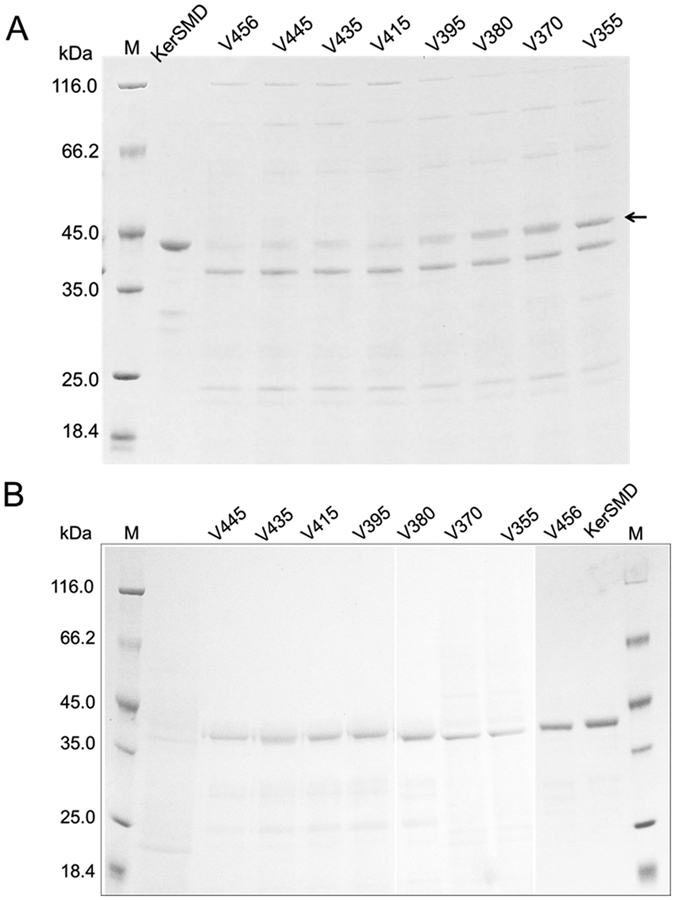
SDS-PAGE analysis of the expression of C-terminal deletion variants in fermentation supernatant (**A**) and molecular sizes of purified keratinases (**B**). M means the standard molecular weight markers. The purified KerSMD was used as a control in picture A. Arrow indicates the lane place of variants.

Deletions were from 7 to 112 residues at the C-terminus, 2.6 to 24% of the total length of KerSMD. The C-terminal truncated derivatives were expressed in *E. coli*. After three days of fermentation, keratinolytic supernatant was detected and collected for purification. The SDS-PAGE showed that KerSMD and variants were about 42 kDa (Fig. 2A). Though the C-terminus was partially truncated, there was no obvious difference in molecular sizes of those variants. However, the secretion levels of keratinases increased significantly from variant V456 to V355. Deletion of the entire C-terminal domain contributed to the highest protein concentration in extracellular broth of V370 variant (Fig. 2A). The purified keratinase derivatives were also analyzed by SDS-PAGE (Fig. 2B). KerSMD displayed a molecular size of 42 kDa, a litter bigger than other variants which were ranged from 40 kDa to 42 kDa.

We have learned that partial truncation of the C-terminus could greatly increase keratinase secretion while minimally affecting molecule size. We hypothesize that the C-terminal domain may inhibit the function of outer membrane proteins for secretion. Similar reports have also shown that C-terminal truncation could increase expression rate of proteases^17^,^19^. Since the PPC domain has many hydrophobic residues, such as Phe and Leu, the increased affinity to bind to hydrophobic membrane proteins or lipids may decline extracellular transport of keratinase^22^. Besides, the unexpected migration of protein bands on the SDS-PAGE may be related to the covalently linked N-propeptide, which was not auto-processed during enzyme maturation and increased keratinase molecule sizes. This deduction was according to our previous study that C-terminal truncation could inhibit N-terminal processing^23^.

### Effects of truncation on substrate specificity and kinetic parameters

The wild type KerSMD and C-terminal deletion variants showed different enzyme activities on macromolecule and micromolecule substrates. As shown in Table 1, all enzymes had proteolytic activities to both casein and feather, indicating that either partial or complete truncation of C-terminal domain had little influence on protein folding. We infer that the C-terminus is an independent domain which is related to substrate specificity. Increasing the number of amino acid deletions led to enhancement of caseinolytic activity and catalytic efficiency (*k*_*cat*_) of variants V415 and V355. The truncation of β-fold6 did not decrease caseinolytic and keratinolytic activities too much. However, losing more than two β-folds (β5 and β6) resulted in a substantial decrease of keratinolytic activity of feather. We deduce that β5 probably is an important structure to the PPC domain in order to maintain spatial integrality or bind keratin substrate. Truncating half of the β-folds resulted in low caseinolytic activities of variants V435 and V415. This may be related to protein conformational changes because the high substrate binding ability (low value of turnover, *K*_*m*_) did not significantly enhance catalytic efficiency as shown in Table 1. Certainly, the exposure of hydrophobic amino acids can also block pocket to receive substrates. In summary, C-terminal deletion was beneficial for enhancing catalytic efficiency to casein and AAPF and the whole truncation of C-terminal domain contributed the highest caseinolytic activity and *k*_*cat*_ /*K*_*m*_ value to V355. All the results indicate that C-terminal domain is important for substrate specificity.

**Figure 3:**
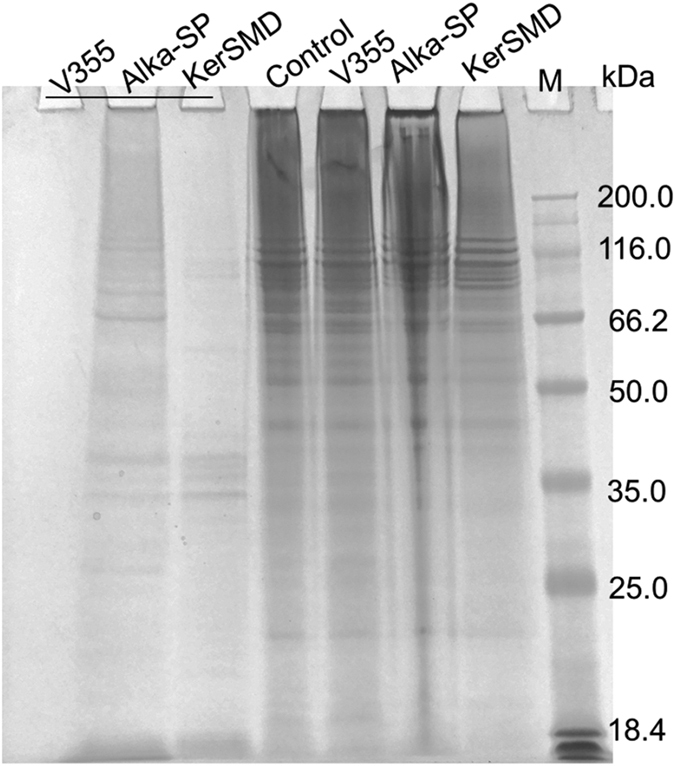
SDS-PAGE analysis of the digestion of keratinase KerSMD, mutant V355, and alkaline serine protease (Alka-SP) on insoluble type I collagen fiber (bovine achilles tendon). The reaction supernatant of three samples was showed with line, and the other reaction mixtures including the control were totally loaded for SDS-PAGE analysis. The enzymes used to digest collagen (1 mg) were about 50 U keratinolytic activity and the incubation was at 37 °C for 12 h.

Since collagen-degrading ability of KerSMD may prevent its application in leather industry, it is important to change substrate specificity by protein engineering. From our previous work, we have found that PPC domain of keratinase KerSMD shared high identity to the vEP C-ter 100, which has the function of binding to collagen^22^,^24^. So, we deduce that collagenase activity of KerSMD may be related to PPC domain. The SDS-PAGE analysis of collagen degradation was conducted to KerSMD, C-terminal deletion mutant V355, and alkaline serine protease (Fig. 3). It showed that alkaline serine protease from *Bacillus licheniformis* had the highest ability to digest insoluble collagen, and KerSMD came second. The soluble protein in the supernatant mainly appeared in the reaction mixture of alkaline serine protease (Fig. 3). There was hardly any digestion of collagen by the C-terminal deletion variant V355. KerSMD was similar to the major extracellular protease of the nosocomial pathogen *Stenotrophomonas maltophilia* which showed obvious activity to degrade collagen and damage skin^25^. Deleting the C-terminal domain might overcome the disadvantage of KerSMD to broaden its application in the food and leather industry. In addition, the ideal protease in leather treatment always needed keratinolytic activity but no collagenase activity for dehairing, and our mutants may be suitable for that application^26^.

### Effects of truncation on thermostability

**Figure 4:**
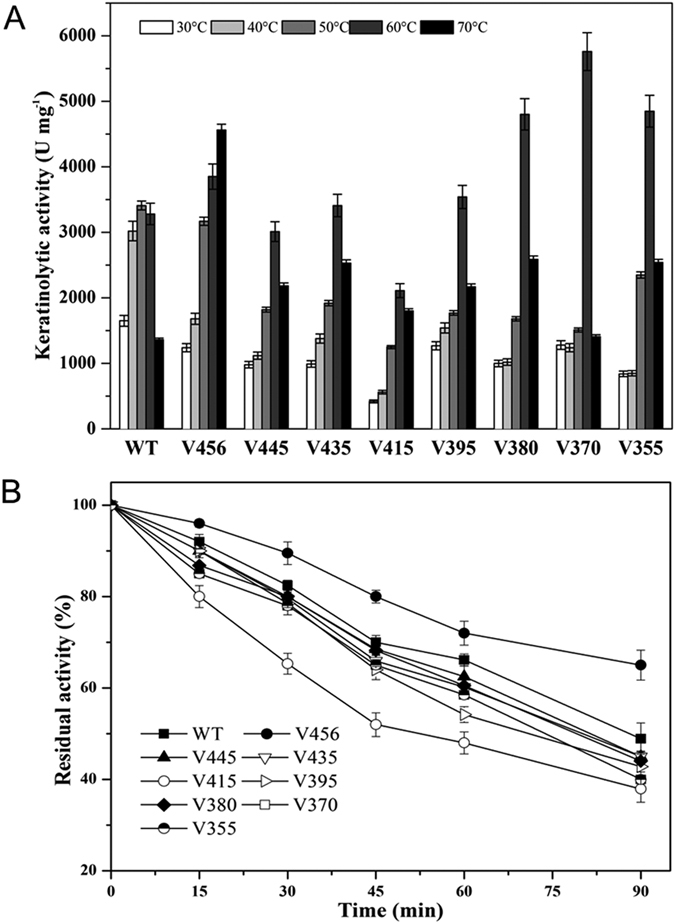
Optimal reaction temperature and thermostablity of wild type (WT) keratinase and its deletion variants. (**A**) Optimal reaction temperature (30–70 °C) of KerSMD and variants; (**B**) Residual activities of KerSMD and variants incubated at 50 °C for different times.

The PPC domain of protease was always characterized as an important factor in improving enzyme thermostability. In order to evaluate whether the PPC domain of KerSMD has similar functions, we further studied the temperature stability and sensitivity of the wild type and its truncated variants. As shown in Fig. 4A, partial deletion of C-terminal domain decreased enzyme activity of keratinase at 50 °C but significantly increased its activity at 60 °C. Variant V370, which had the entire truncation of β-folds of the C-terminal domain, obtained the highest keratinolytic activity of 5760 U mg^−1^ at 60 °C, about a 1.7-fold improvement compared to the wild type (3280 U mg^−1^). It seemed that the deletion of the C-terminal domain contributed to the keratinase thermophilic property. However, increasing the temperature resulted in enormous decreases of all mutants except for V456 which showed the highest activity at 70 °C. We deduce that β-fold6 may be a potential repressor of enzyme catalysis.

The keratinase thermostability at 50 °C was also analyzed (Fig. 4B). It seemed that truncation of the C-terminal domain always had a disadvantage on enzyme stability. The variant V415, a half truncation of the C-terminal domain, showed the worst stability, which was a 10% decline of enzyme activity when compared with the wild type. However, all variants still showed more than 40% residual activity after a 90-minute incubation. The variant V456 improved enzyme stability by about 20% at the end of heat inactivation process (Fig. 4B).

The C-terminal extension was common in amylase, and its truncation can enhance enzyme thermal stability^27^. However, the deletion of the C-terminal domains of halophilic protease MCP-03 decreased thermal stability^18^. The hyperthermostable protease Tk-SP also required a C-terminal domain for hyperstability^17^. Though our results showed that the deletion of the PPC domain of keratinase KerSMD increases its thermophilicity but may reduce thermal stability, a partial truncation was interesting to improve enzyme stability. We could apply thermophilic but instable keratinase mutants for some special processes which need enzyme inactivation after food treatment.

### Effects of truncation on alkaline and salt tolerability

**Figure 5:**
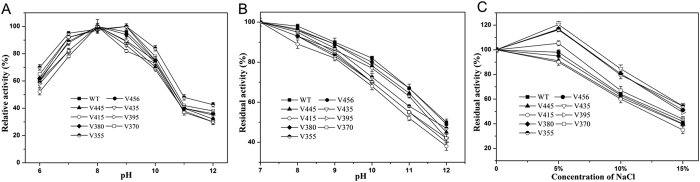
Optimal reaction pH and tolerance of alkalinity and salt of wild type (WT) keratinase and its deletion variants. (**A**) Optimal reaction pH (6.0–12.0) of KerSMD and variants; (**B**) Alkaline tolerance (pH 7.0–12.0) of KerSMD and variants; (**C**) KerSMD and variants in the presence of different concentrations of NaCl (0–15%, w/v). The activities of KerSMD and variants were dependent on casein substrate.

The keratinase KerSMD is an alkaline protease and we deduced that the PPC domain may be related to enzyme alkalophilicity. It showed that partial truncation of the PPC domain resulted in activity decrease at pH 9.0–12.0 (Fig. 5A). However, the complete deletion of the PPC domain enhanced the basophilicity of variant V355, which showed more than a 40% increase in relative activity at a pH of 12.0. Compared to the wild type keratinase, the variant V355 also improved its activity by about 10% in neutral solution (pH 7.0). Though the truncation of the PPC domain decreased alkaline tolerability, variants V355 and V456 had more than 50% residual activity, approaching the value of wild type (Fig. 5B). Since the processes of feather degradation and leather dehairing were always in an alkaline environment, the high performance of alkaline keratinase could be more meaningful^26^,^28^.

Anti-salt or halotolerance is an important characteristic to evaluate whether the enzyme can be used in industrial processes^18^,^29^. We used different concentrations of NaCl to monitor the halotolerance of KerSMD and its different variants (Fig. 5C). Variants V395, V380, V370, and V355 all tended to be halophilic keratinases whose activities were enhanced by 5% (w/v) NaCl. Those four mutants all showed higher residual activity than the wild type and other mutants in high concentration saline solution. The variant V355 obtained the highest tolerability to salt, nearly 60% activity in 15% (w/v) NaCl solution. The halophilic phenomenon might relate to the Asp and Glu residues that could resist electrolytes^30^. Comparison of the sequences of halophilic and non-halophilic variants showed that the ratio of Asp and Glu residues was larger in halophilic variants (Fig. S1). Suitable control of the ratio of negatively charged residues by C-terminal truncation probably was useful to enhance salt tolerability. The increase of negatively charged residues on protein surface may also be responsible for the formation of highly ordered shells of water molecules that prevent denaturation under high alkalinity^30^.

### Effects of truncation on chaotropic agents and detergent tolerability

**Figure 6:**
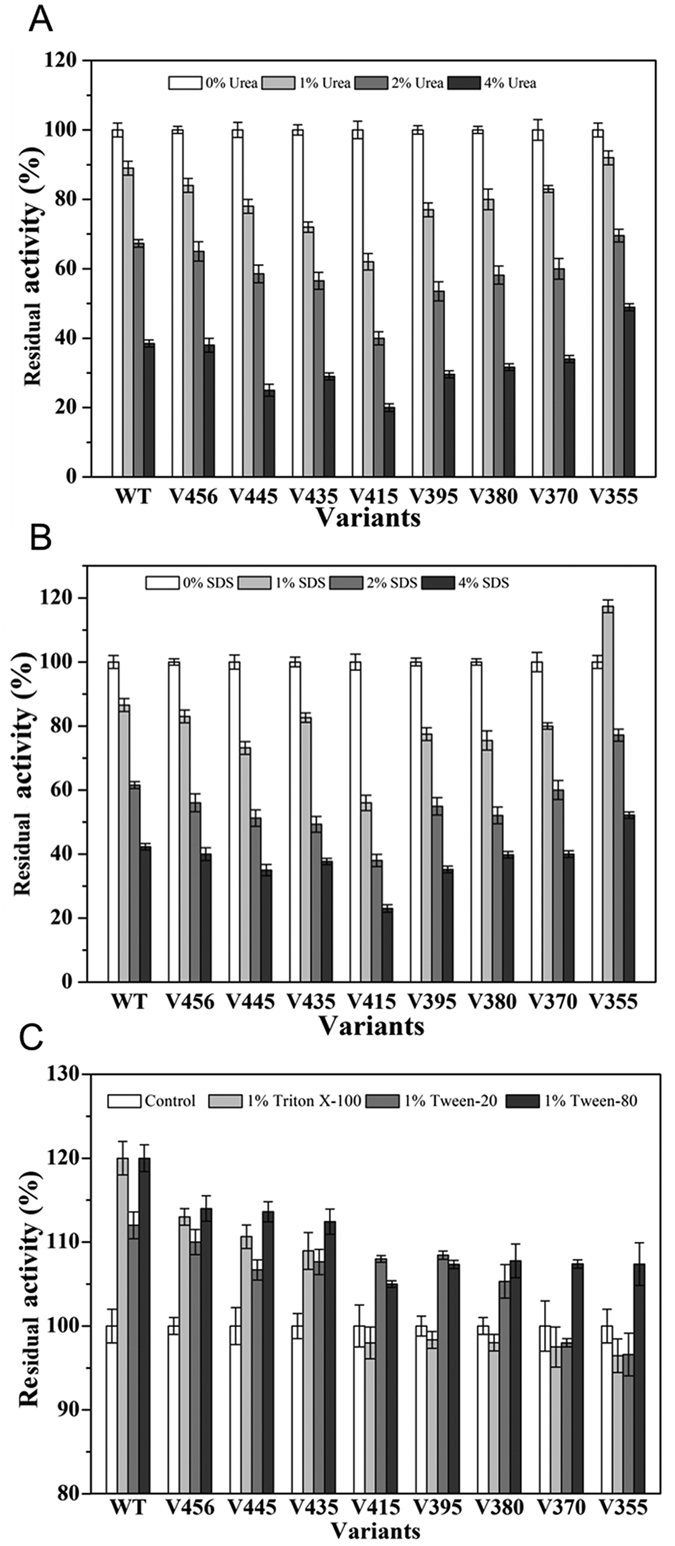
Effects of chaotropic compounds on wild type (WT) keratinase and its deletion variants. (**A**) KerSMD and variants in the presence of different concentrations of SDS (0–4%, w/v); (**B**) KerSMD and variants in the presence of different concentrations of urea (0–4%, w/v); (**C**) KerSMD and variants in the presence of 1% (v/v) Triton X-100, Tween 20, and Tween 80. The activities of KerSMD and variants were dependent on AAPF substrate. Relative activity was a calculated as percentage of the activity of the control as 100%.

Keratinase is a potential protease used in laundry detergents that usually contain high concentrations of surfactants, such as SDS, Triton X-100, Tween-20, and Tween-80^20^,^28^. Those surfactants and chaotropic agents have great influence on protein structure. So enzyme stability in different surfactants and chaotropic agents is an important parameter of commercial enzymes^9^,^31^. We investigated the effect of many agents (urea, SDS, Triton X-100, Tween 20, and Tween 80) on keratinases. As shown in Fig. 6A,B, enzyme activity was destroyed by increasing concentrations of chaotropic compounds, and the truncation of C-terminal domain further decreased enzyme stability in chaotropic solution. The variant V415 showed the lowest stability to urea and SDS, with a nearly 80% decline of activity (Fig. 6). However, the variant V355 obtained significant tolerability to chaotropic compounds, and reserved more than 40% activity in 4% (w/v) urea or SDS. Remarkably, the addition of 1% (w/v) SDS to variant V355 improved its enzymatic activity, similar to a surfactant-stable keratinase from *Streptomyces aureofaciens* K13^32^. Though site-saturation mutagenesis and recombination of subtilisin E obtained excellent mutants that had 40–60% activity in 4% (w/v) SDS^31^,^33^, it might be more time-consuming than our C-terminal truncation strategy.

On the contrary, the other surfactants, Triton X-100, Tween-20, and Tween-80, had positive effects on enzymatic activities. From Fig. 6C we could learn that the wild type keratinase had the highest enzyme activity (approximately 120% activity) in the presence of those surfactants. It showed that surfactants had the ability to activate keratinase to catalyze substrate. In addition, we could also see that C-terminal truncation weakened surfactant tolerability of keratinase (Fig. 6C). The surfactant can break hydrogen bonds or salt bridges to unfold protein^33^. Our results showed that the PPC domain probably could strengthen molecular interaction and enhance stability to the surfactant.

### Structure modeling and analysis of wild type keratinase and mutants

**Figure 7:**
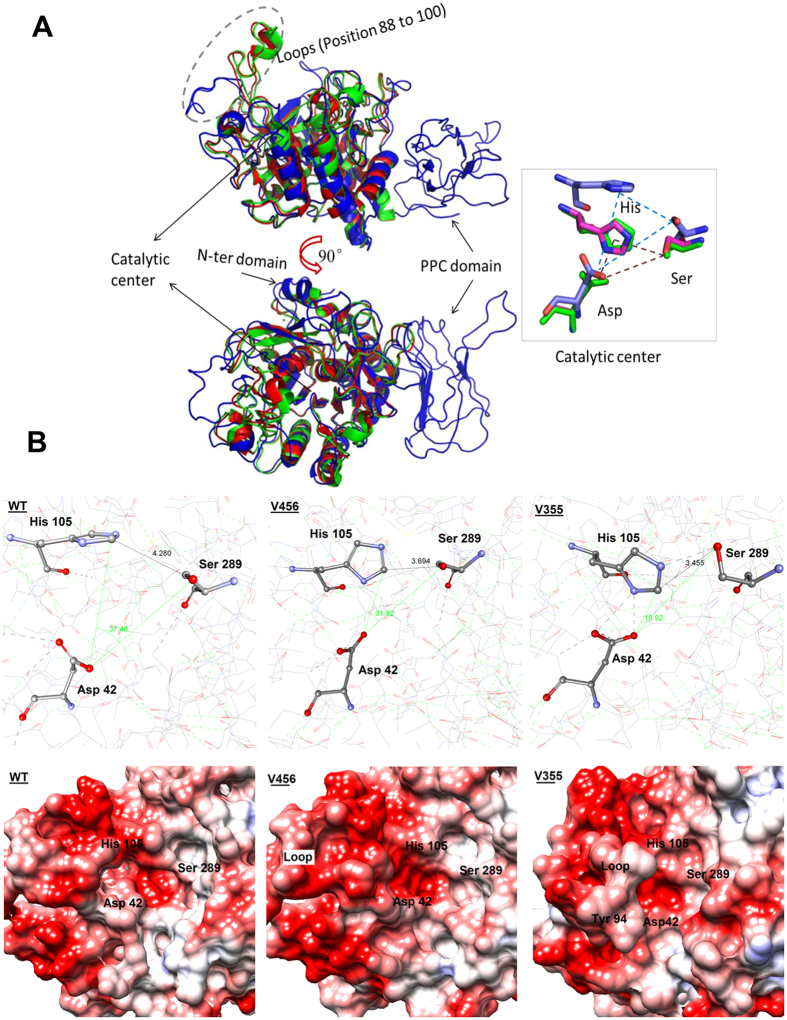
Structure alignment (**A**) of modeled KerSMD (blue) with other high homology structures 3LPA (red) and 3AFG (magenta), and model structures (**B**) of wild type KerSMD and its variants V456 and V355. The catalytic triangle area of KerSMD is blue dashes while 3LPA and 3AFG are brown. The hydrogen bonds around triangle catalytic center are displayed by green dashes while the hydrogen bonds linked to catalytic residues (Asp42, His105 and Ser289, shown in ball and stick) are shown by black dashes. The distance between His105 and Ser289 is labeled with a black line. The angle of those three residues is displayed with a green line. Electrostatic surface was drawn with the software Chimera 1.9 (UCSF Computer Graphics Laboratory, USA). The negative potential is red, neutral was white, and positive is blue.

From the NCBI blasting results of the Protein Data Bank (PDB) database, we found that the KerSMD sequence shared the highest identity (47%) with the subtilisin-like proteases (PDB no. 3LPA, 3LPC, 3LPD, 3TI9, and 3TI7), a 37% identity with thermostable serine protease (PDB no. 1DBI), and a 35% identity with thermitase (PDB no. 1THM). Though the above proteins showed high identity, their structures are all lack of the C-terminal domains except for *Thermococcus kodakaraensis* protease (PDB no. 3AFG, about 25% identity). So we took them as the reference structures for the modeling work of KerSMD. Modelling of the C-terminal domains of KerSMD was based on the templates of vEP C-ter 100 (PDB no. 2LUW, with 83% query coverage and 30% identity). We also did sequence alignment of those reference structures to show their similarity in supplementary materials Fig. S1. We can see that catalytic domains were highly conserved. For example, the amino acid sequences from position 110 to 165, 176 to 188, and 210 to 240 all showed more than a 5-point conservative factor. The C-terminal domain of KerSMD (from position 370 to 480) was lower conserved. So we did the modeling work of C-terminal domain according to 2LUW independently. The structure alignment was conducted by PyMol software as shown in supplementary Fig. S2 and Fig. 7. We found that the KerSMD model was highly similar to 3LPA, 3LPC, 3LPD, 3TI9, 3TI7,1DBI, 1THM, and 3AFG. Their carbon backbones showed high conservation because those proteins all have a subtilisin-like catalytic domain (a catalytic peptidase S8). The major differences were the unordered loops and the independent PPC domain. In our former reports^23^, we have discussed that the PPC domain and loops may be related to the substrate specificity. We also aligned the catalytic triangle residues Asp, His, and Ser (Fig. 7A). The catalytic cleft of the modelled structure was larger than the original references, indicating that KerSMD probably tended to hydrolyze macromolecule substrates, such as insoluble keratin.

Variants V456 and V355 showed high thermostability and catalytic activity, respectively. In order to compare the structure changes between the wild type keratinase and variants, we did structure modeling. The differences in catalytic center were showed in Fig. 7B. Since the keratinase KerSMD is a serine subtilisin-like protease, its proteolysis also depends on three important amino acids (Asp42, His105, and Ser289) in the catalytic center^23^. We found that the distances between Ser289 and His105 in variants V456 and V355 were shorter than the wild type. In addition, the angle from Asp42 to His105 and Ser289 was also decreased after the C-terminal truncation, forming a smaller volume for the catalytic center. To some extent, the shorter distance between His105 and Ser289 may accelerate the speed of electron transport in the hydrolysis reaction, which probably improves catalytic activity of V355 on synthetic substrate AAPF. Though the C-terminal truncation could increase catalytic activity, it diminished protein stability to high temperatures. We found that the triangle catalytic center of V355 had more hydrogen bonds than the wild type and V456. The V456 with improved thermostability showed the lowest amounts of hydrogen bonds. The increased amounts of hydrogen bonds may reduce the flexibility of active residual which is important to bind to substrate and provide electron acceptors^34^.

We also predicted the surface electrostatic potential around the catalytic center in Fig. 7 by Chimera 1.9. The red was the main color of protein surface, indicating that the catalytic domains of KerSMD and its mutants had a negative potential. From the study of the electrostatic potential of the original reference structures (Fig. S3), we also learned that KerSMD surface had more negative potential. It has been reported that negative potential is probably related to detergent tolerability of subtilisin E^31^,^33^. Since keratinase KerSMD has a similar catalytic domain and center to subtilisin protease, the increase of negative potential can also improve enzyme tolerability to SDS or other detergents. In this work, our mutants V355 showed the significant enhancement of SDS tolerability. Except for the negative potential, we inferred that the flexible loop above the catalytic center may also have an important effect on detergent tolerability. Comparing the mutant V355 with the wild type and V456, we found that the weak negative loop was shifted from left to right (Fig. 7), which possibly protected the catalytic sites against anion agents such as SDS. The active loop was also reported in another protease AprV2 (Protein Data Bank: 3LPA, 3LPC, and 3LPD) which needs a special site to bind to the substrate^35^. Though the mechanism of SDS tolerability of proteases is still unclear, it has been reported that changing the substrate binding ability can improve enzyme adaptability in detergent solution^31^. However, more work should be conducted to prove that inference.

## Conclusion

In summary, a simple technology of protein engineering was introduced to enhance catalytic efficiency, thermophilicity, anti-salt and detergent tolerance of keratinase KerSMD. In this work, we partially truncated the PPC domain of keratinase KerSMD according to the distribution of six β-folds, and several variants were obtained. The variants V435, V415, V395, V380, V370, and V355 showed improved catalytic efficiency to synthetic substrate AAPF, and the V355 obtained the highest value (143.6 s^−1^ mM^−1^). Though the truncation resulted in a small decline of keratinolytic activity, the variants V370 and V355 still had high protease activity to hydrolyze casein and feather substrate. The variant V355 could be potentially used for laundry detergent and leather treatment because of its low collagenase activity, thermophilicity, and high tolerance to alkalinity, salt, chaotropic agents, and detergent. Our work not only provided a simple technology of protein engineering but also complemented the function of the PPC domain of KerSMD.

## Materials and Methods

### Bacterial strains and growth conditions

The gene encoding keratinase KerSMD was cloned from the *Stenotrophomonas maltophilia* BBE11-1 genome (China Center for Type Culture Collection M2012495) by PrimeSTAR HS DNA polymerase (TaKaRa, Dalian, China). The strain *Escherichia coli* BL21 (DE3) and the plasmid pET22b were purchased from Novagen (Darmstadt, Germany). The strains were cultured at 37 °C in Luria-Bertani (LB) medium with 100 μg ml^−1^ ampicillin. After OD_600_ reached 0.6, the temperature was shifted to 20 °C and isopropyl-β-D-thiogalactoside (IPTG) was added to a final concentration of 0.2 mM for induction.

### DNA cloning and vector construction

The amplification of DNA sequences used specific oligonucleotide primers with NcoI and XhoI restriction sites (Table S1). Two restriction endonucleases, NcoI and XhoI, were used to digest the gel-purified PCR products as well as the pET22b vector. Then ligation was conducted with T4 DNA ligase at 16 °C overnight. The mixture was directly transferred into competent *E. coli* BL21 (DE3) cells. The bacterial solutions were coated on LB solid medium. After incubation for 12 h at 37 °C, a single colony was chosen for verification and expression. The monoclonal colony PCR and DNA sequencing were used to verify correct mutants. All mutants used the signal peptide of the *pelB* leader to make extracellular secretion of recombinant keratinases.

### Purification of keratinases and SDS-PAGE analysis

After 72 h of fermentation, the culture supernatant was harvested by centrifugation at 7, 000 × g. The purification procedures of recombinant keratinases were conducted as previously described by Fang *et al*. which was processed with two columns, HiTrap^TM^ Phenyl FF and Q FF, and an AKTA pure (GE Healthcare, Sweden)^22^. Fractions with protease activity were collected, analyzed, and stored at −80 °C.

The sodium dodecyl sulfate polyacrylamide gel electrophoresis (SDS-PAGE) was carried out as previously described by King and Laemmli^36^. The kit for SDS-PAGE preperation were purchased from Beyotime (Shanghai, China) and the procedure of preperation was according to product description. The samples (1 μg) were loaded onto the SDS-PAGE gel for running. The protein marker was also purchased from Beyotime and 10 μl was used for loading onto the gel.

The SDS-PAGE analysis related to the degradation of insoluble type I collagen fiber (bovine achilles tendon, Worthington Biochemical Co., USA) was conducted. The collagen (1 mg) was treated with different enzymes (50 U keratinolytic activity) at 37 °C for 12 h in 50 mM glycine-NaOH buffer (pH 9.0). The wild type keratinase, variant V355, and alkaline serine protease (from *Bacillus licheniformis*, Amano Enzyme, Japan) were used to degrade collagen. After centrifugation (12, 000 × g, 5 min, 4 °C), the supernatant liquid of reaction mixture and precipitate were mixed with loading buffer (98 °C, 5 min) and loaded to the gel, respectively.

### Keratinolytic and caseinolytic activity assay

The assays of enzyme activities were performed as previously described by Fang *et al*. with minor modifications^21^. Keratinolytic activity was detected using feather meal from a local chicken farm, while caseinolytic activity was measured using casein as the substrate. The enzyme-substrate mixture was incubated at 50 °C for 20–60 min. Then, the Folin-phenol reagent (Sangong, Shanghai, China) method was used to measure the release of tyrosine. One unit of enzyme activity is corresponding to a 0.001 AU min^−1^ ml^−1^ increase in absorbance at 660 nm. Protein concentration was determined by the Bradford protein assay reagent kit (Beyotime). The specific activity was defined as the total activities of per mg keratinase. All experiments were repeated three times and the standard deviation was calculated.

### Measurement of kinetic parameters of synthetic peptide

The measurement of kinetic parameters was determined as previously described by Windhorst *et al*.^25^. Enzyme reactions with synthetic *p*-nitroanilide peptide of succinyl-Ala-Ala-Pro-Phe-*p*NA (Sigma-Aldrich, USA) were conducted in 5% v/v dimethylformamide in 100 mM Tris-HCl buffer (pH 8.2) at 25 °C. The absorbance at 405 nm (ε_405_ = 9600 M^−1^ cm^−1^) was recorded within initial time intervals by using a UV2450 spectrophotometer (Shimadzu, Japan). The kinetic parameters *K*_*m*_ and *k*_*cat*_ were calculated by nonlinear regression analysis. The various assays were repeated at least twice and the standard deviation was calculated.

### Effect of temperature on enzyme activity and stability

The effects of temperature on keratinase activity and stability were determined at a stable pH value (50 mM glycine-NaOH buffer, pH 9.0). Enzyme activities at different temperatures (ranging from 30 °C to 70 °C) were recorded. The thermal stabilities of various variants and wild type KerSMD at 50 °C were determined by incubation at different time intervals. We used the mixture of inactive keratinases and substrates as a control. The initial activity without enzyme inactivation was considered as 100% and the residual activities were calculated as a percentage of this initial value.

### Effect of pH on enzyme activity and stability

The optimal pH for the reaction and alkaline stability of the wild type and variants were assayed. The 100 mM buffers for different pH values are the following: Na_2_HPO_4_-NaH_2_PO_4_ for pH 6.0 to 8.0, Tris-HCl for pH 8.0 to 9.0, and glycine-NaOH for pH 9.0 to 12.0. After overnight incubation with different buffers (pH 7.0–12.0) at 4 °C, the alkaline stability was determined. We used the mixture of inactive keratinases and substrates as a control. The highest enzyme activity was considered as 100%, and other values were recorded as a percentage of the highest value.

### Effects of salt, chaotropic agent, and detergents on enzyme stability

The effects of salt, chaotropic agents, and detergent on enzyme stability were assayed. We used different concentrations of NaCl (0%, 5%, 10%, 15%, w/v) in 50 mM glycine-NaOH buffer (pH 9.0) with feather meal as the substrate. The chaotropic agents were urea and SDS (sodium dodecyl sulfonate), and the concentrations ranged from 0% to 4% (w/v). The other detergents (1% v/v) used to determine enzyme stability were Triton X-100, Tween-20, and Tween-80. Since chaotropic agents and detergents are special compounds, which can dissolve casein protein and influence protease activity detection, the synthetic substrate AAPF was used to assay their effects on enzyme stability. We used the mixture of inactive keratinases and substrates as a control. The highest enzyme activity was considered as 100%, and other values were recorded as a percentage of the highest value.

### Model constructions of wild type and mutants V456 and V355

The homology models of KerSMD and mutants V456 and V355 were constructed with the program Modeller V9.11. The catalytic domain model was based on crystal structures of subtilisin-like proteases (Protein Data Bank: 3LPA, 3LPC, 3LPD, 3TI9^37^, 3TI7^37^, 1DBI^38^, 1THM^39^, 3AFG^17^), and the lowest score model was chosen. We did the energy minimization and fully reduced the steric clashes of model protein by molecular dynamics (MD) simulations with NAMD software (http://www.ks.uiuc.edu/). The CHARMM force field, periodic boundary condition of water box, and Particle Mesh Ewald (PME) algorithm were employed. By setting a constant temperature of 310 K, 1 atm pressure, and 1 ns running time, the whole system was fully relaxed to obtain the minimum energy models. The online tools of PROCHECK, ERRAT of SAVES (http://services.mbi.ucla.edu/SAVES/) showed the stereochemical quality value to be 95%. We also predicted the electrostatic surface of the optimal model protein by Chimera 1.9 (http://www.cgl.ucsf.edu/chimera/).

### Statistical analysis

All experiments were performed at least three times. Statistical analysis based on the SPSS software was conducted to evaluate the effects of different factors (temperature, pH, salt, chaotropic agent, and detergents) on enzyme activities. The statistical evaluation on the differences on the enzyme activities of the variants and the WT was also included. The final results were expressed as the mean ± standard deviation and tested by Student’s t-test (p < 0.05).

## Additional Information

**How to cite this article**: Fang, Z. *et al*. Improved catalytic efficiency, thermophilicity, anti-salt and detergent tolerance of keratinase KerSMD by partially truncation of PPC domain. *Sci. Rep*. **6**, 27953; doi: 10.1038/srep27953 (2016).

## Supplementary Material

Supplementary Information

## Figures and Tables

**Table 1:** Enzyme activities and kinetic parameters of KerSMD and its C-terminal deletion variants.

Enzyme	Activities of different substrates (U mg^−1^)	Kinetic parameters of AAPF			
	Casein	Feather	*K*_*m*_ (mM)	*k*_*cat*_ (s^−1^)	*k*_*cat*_ /*K*_*m*_ (s^−1^ mM^−1^)
KerSMD	3779 ± 20	3409 ± 56	0.66 ± 0.04	46.0 ± 0.4	71.0 ± 5.2
V456	3430 ± 12	3170 ± 110	0.74 ± 0.15	48.0 ± 0.2	65.0 ± 1.4
V445	3610 ± 32	1820 ± 78	0.78 ± 0.05	50.2 ± 0.7	64.2 ± 2.5
V435	850 ± 15	1920 ± 75	0.60 ± 0.05	55.8 ± 0.9	94.2 ± 0.3
V415	320 ± 10	1250 ± 59	0.54 ± 0.02	58.3 ± 0.5	108.3 ± 0.2
V395	2510 ± 38	1770 ± 112	0.70 ± 0.03	78.0 ± 1.4	110.8 ± 1.0
V380	3020 ± 27	1680 ± 83	0.76 ± 0.08	101.2 ± 1.4	133.8 ± 1.2
V370	4570 ± 25	1510 ± 54	0.73 ± 0.06	94.2 ± 0.6	129.0 ± 1.9
V355	5340 ± 23	2350 ± 62	0.86 ± 0.04	123.5 ± 0.4	143.6 ± 2.2
