# Supplementary material for: Improved catalytic efficiency, thermophilicity, anti-salt and detergent tolerance of keratinase KerSMD by partially truncation of PPC domain
Source: Sci Rep. 2016 Jun 14;6:27953. doi: 10.1038/srep27953 (PMC4906391; doi:10.1038/srep27953)
Supplement: Supplementary Information [file srep27953-s1.pdf]

# Supplementary materials

for

**Improving catalytic efficiency, thermophilicity, anti-salt and detergent tolerance of keratinase KerSMD by partially truncation of PPC domain**

**Zhen Fang [1, 2, 5], Juan Zhang [1, 5] *, Guocheng Du [3, 5] *, Jian Chen [4, 5]**

[1] *Key Laboratory of Industrial Biotechnology, Ministry of Education, Jiangnan University, Wuxi 214122, China*

[2] *Synergetic Innovation Center of Food Safety and Nutrition, Wuxi 214122, China*

[3] *Key Laboratory of Carbohydrate Chemistry and Biotechnology, Ministry of Education, Jiangnan University, Wuxi 214122, China*

[4] *National Engineering Laboratory for Cereal Fermentation Technology, Jiangnan University, Wuxi 214122, China*

[5] *School of Biotechnology, Jiangnan University, Wuxi 214122, China*

*Corresponding authors: Juan Zhang, E-mail: zhangj@jiangnan.edu.cn; Guocheng Du, E-mail: gcdu@jiangnan.edu.cn. Tel.: +86-510-85918307, Fax: +86-510-85918309; Fax: +86-510-85914371.

School of Biotechnology, Jiangnan University, 1800 Lihu Road, Wuxi 214122, China.

**Table S1.** Oligonucleotide primers used for gene cloning of KerSMD and its C-terminal deletion mutants.

| Primer name | Primer sequence (5' to 3') |
|---|---|
| F-kerSMD | ACATG*CCATGG*CCGGGTTGCCGACCC |
| R-kerSMD | CCG*CTCGAG*TTACTGCGTGGCGAGGATGCTC |
| V456 | CCG*CTCGAG*TTACGCTTCGCCCACCACC |
| V445 | CCG*CTCGAG*TTATGCCACTGGCTTGTTCACC |
| V435 | CCG*CTCGAG*TTAGGACGTGCCGGGACGG |
| V415 | CCG*CTCGAG*TTAGGCGATGTAGACGGACACGTT |
| V395 | CCG*CTCGAG*TTAAGCGGCTGCCTCGAAGCT |
| V380 | CCG*CTCGAG*TTAGTTCAGTCCCCCCACCGC |
| V370 | CCG*CTCGAG*TTACAGCGGCGTCGCCACC |
| V355 | CCG*CTCGAG*TTACAGTGCCTTGGCCAGCG |

[a] The italic letters represent the sites for restriction digest.

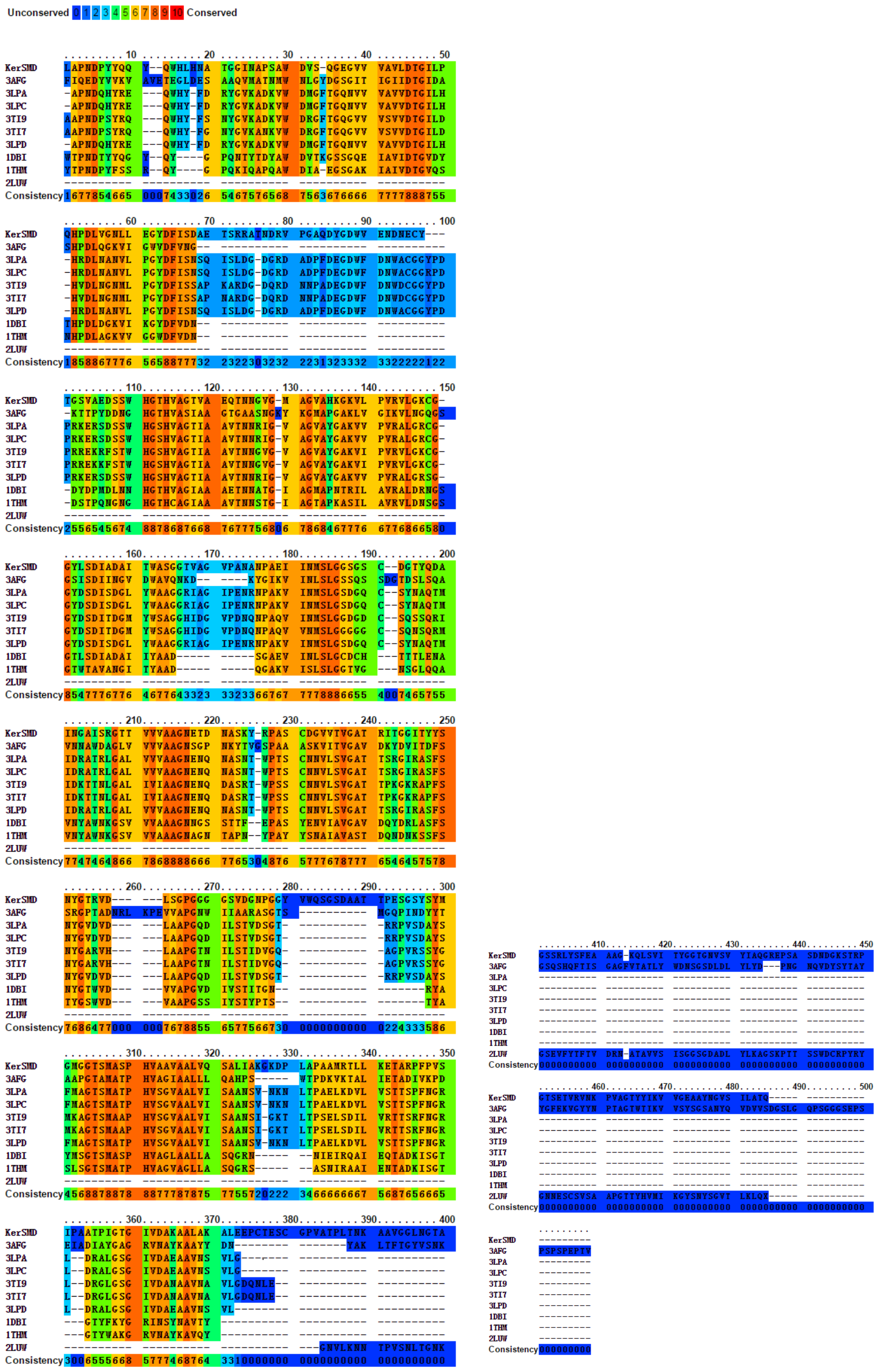


**Fig. S1.** Sequence alignments of KerSMD and other high homology proteins (PDB no. 3LPA, 3LPC, 3LPD, 3TI9, 3TI7,1DBI, 1THM, 3AFG, and 2LUW).

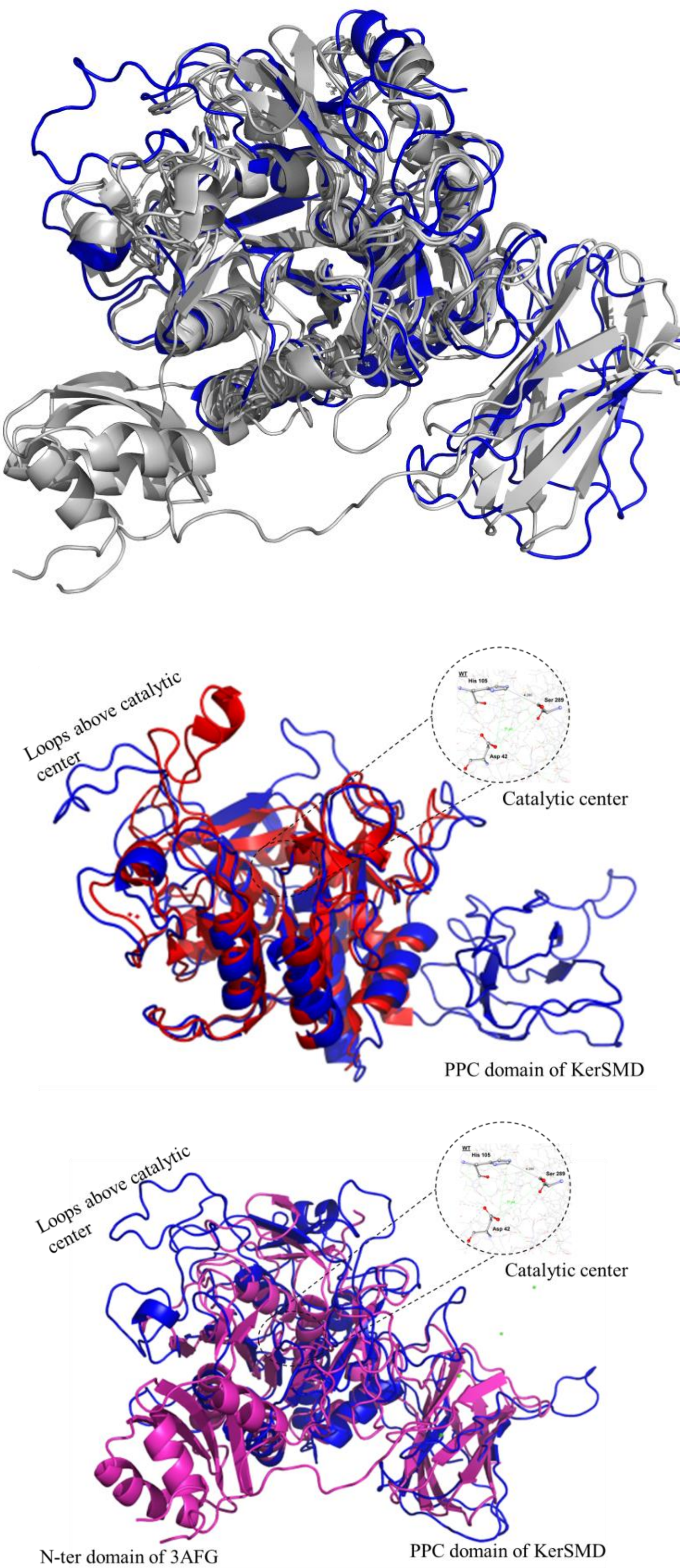


**Fig. S2.** Structure alignment of the modeled KerSMD (blue) and other high homology proteins (PDB no. 3LPA, 3LPC, 3LPD, 3TI9, 3TI7,1DBI, 1THM, and 3AFG). The structures 3LPA (red) and 3AFG (magenta) were respectively studied. The major differences are the loop direction and the PPC domains.

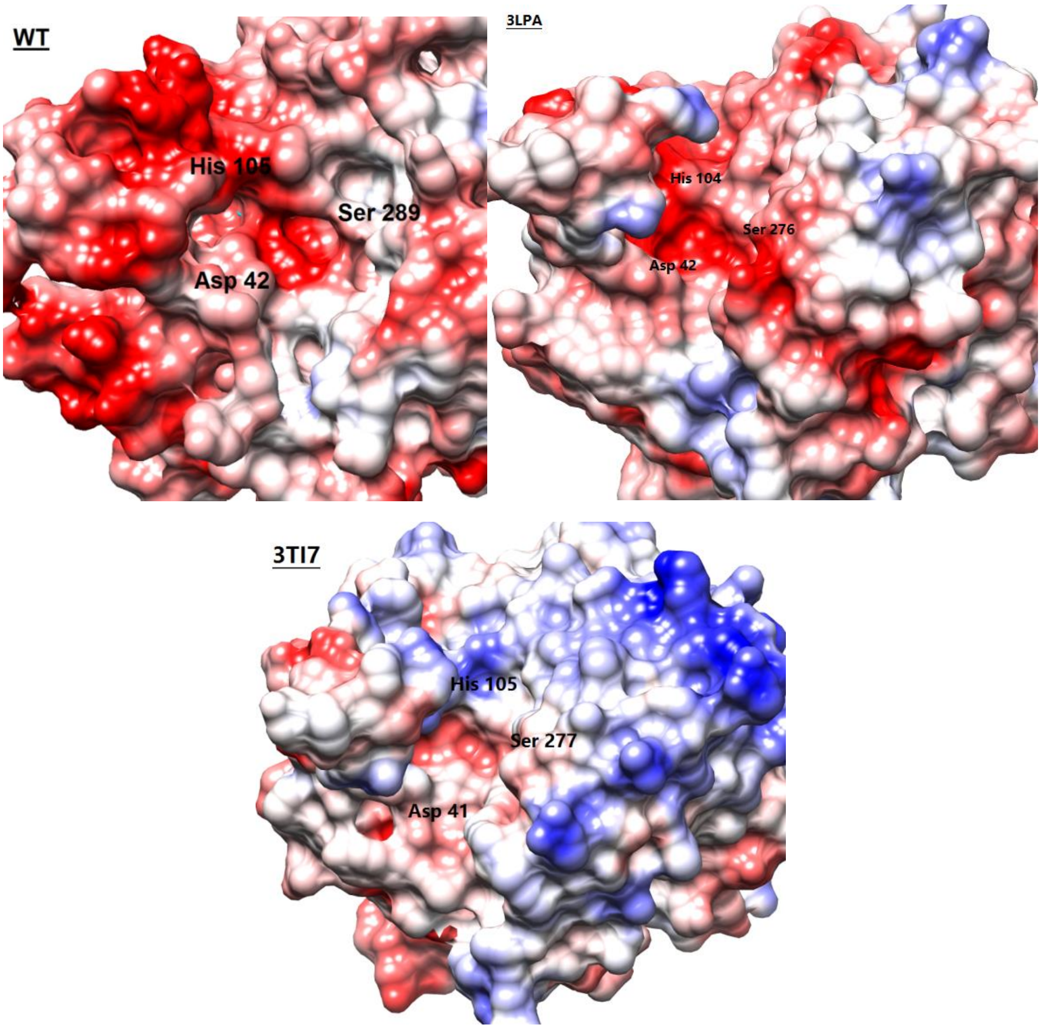


**Fig. S3.** Electrostatic potential surface of catalytic domains of the modeled KerSMD (WT) and reference structures (3LPA and 3TI7). We calculated electrostatic potential according to Coulomb's law, showing color from red for negative potential, to white near neutral, to blue for positive potential. Since 3LPA and 3TI7 have the highest homology to KerSMD, we used these two as reference. From the comparing, we can learn that KerSMD has more negative potential around catalytic triad (Asp, His, Ser). It probably related to anti-salt and basophilicity of KerSMD.
